# The burden of HEV-related acute liver failure in Bangladesh, China and India: a systematic review and meta-analysis

**DOI:** 10.1186/s12889-023-17302-2

**Published:** 2023-11-29

**Authors:** Rui Dong, Dongchun Chang, Zhenghan Luo, Mengting Zhang, Qing Guan, Chao Shen, Yue Chen, Peng Huang, Jie Wang

**Affiliations:** 1 Department of Fundamental and Community Nursing, School of Nursing, Nanjing Medical University, 101 Longmian Avenue, Jiangning District, Nanjing, 211166 Jiangsu China; 2 East China Institute of Biomedical Technology, Nanjing, China; 3 Nanjing Municipal Center for Disease Control and Prevention, Nanjing, China; 4 Department of Epidemiology, School of Public Health, Nanjing Medical University, 101 Longmian Avenue, Jiangning District, Nanjing, 211166 Jiangsu China

**Keywords:** Hepatitis E, Etiology, Prevalence, Mortality, Acute Liver Failure

## Abstract

**Background:**

Hepatitis E can potentially progress to HEV-related acute liver failure (HEV-ALF). East and South Asia bear a substantial burden of HEV infection, with Bangladesh, China, and India facing the most severe threat in this region. Therefore, we conducted a systematic review and meta-analysis to evaluate the burden of HEV-ALF in these three high-risk countries.

**Methods:**

A systematic literature search was performed utilizing PubMed, the Cochrane Library, Medline, Embase, and Web of Science databases. Studies in English or Chinese that reported data on the burden of HEV-ALF in Bangladesh, China and India were included. Outcomes were pooled with meta-analysis utilizing R software. Estimates were calculated with random-effects models, and subgroup analysis and sensitivity analysis were conducted to address heterogeneity. Egger’s test and Begg’s test were performed to assess publication bias.

**Results:**

A total of 20 eligible studies were included in this study. The pooled HEV-attributable proportion of viral-related acute liver failure was estimated to be 40.0% (95% CI: 0.28–0.52), 30.0% (95% CI: 0.18–0.44), and 61.0% (95% CI: 0.49–0.72) among non-pregnant individuals in India, China and Bangladesh, while in Indian pregnant females, it was 71.0% (95% CI: 0.62–0.79). The combined prevalence among non-pregnant HEV-infected participants was 28.0% (95% CI: 0.20–0.37) and 10.0% (95% CI: 0.01–0.28) in India and China, and it was 34.0% (95% CI: 0.27–0.42) in Indian pregnant females with HEV infection. The overall mortality of HEV-ALF was estimated to be 32.0% (95% CI: 0.23–0.42) and 64.0% (95% CI: 0.50–0.77) among the non-pregnant and the pregnant participants in India, and it was 23.0% (95% CI: 0.14–0.34) in Chinese non-pregnant participants.

**Conclusions:**

The burden of HEV-ALF in Bangladesh, China, and India is non-negligible despite geographic and population heterogeneity. The prevention of HEV infection and early recognition of HEV-ALF are of great significance, especially in high-risk countries and populations.

**Registration:**

PROSPERO registration ID is CRD42022382101.

**Supplementary Information:**

The online version contains supplementary material available at 10.1186/s12889-023-17302-2.

## Introduction

Hepatitis E virus (HEV) is a positive-sense single-stranded RNA virus that is one of the leading causative agents for acute viral hepatitis worldwide [1]. A recent meta-analysis indicated that the global prevalence of HEV infection was estimated to be 12.47%, corresponding to approximately 939 million individuals who have experienced past HEV infection [2]. The prevalence of HEV infection varies in different geographical regions. Previous studies have revealed that a high burden of HEV infection exists in East and South Asia, which suffer the most severe threat of HEV infection [2–4]. Among the countries in this geographic region, Bangladesh was among the top three countries with the highest incidence of HEV infection in 2017, followed by India and China [5]. The clinical manifestations of HEV infection are diverse, ranging from asymptomatic to HEV-related acute liver failure (HEV-ALF) [1, 2]. The specific host populations, geographic regions, and HEV genotypes (GTs) differ markedly regarding the clinical presentations of this disease [6]. Although heterogeneity exists, studies have indicated that HEV is still one of the primary contributors to acute liver failure (ALF), causing significant mortality [7–9]. The high prevalence of HEV infection and the significant mortality caused by HEV-ALF highlight the significant public health concern posed by HEV infection.

Several previous studies have indicated that HEV-ALF is usually considered to be underreported because HEV is not a routinely screened etiology in ALF patients. It has also been reported that many HEV-ALF cases are misdiagnosed as drug-induced liver injury or ALF with etiologies other than HEV [10–12]. Thus, HEV-ALF is considered an issue that has long been underestimated. Moreover, the mechanism underlying HEV-ALF progression has not been fully elucidated, and effective treatments have rarely been reported [1, 12, 13]. Current treatment strategies for HEV-ALF usually involve critical care, artificial liver support, and liver transplantation, which often cause high medical expenditures and are usually unavailable in resource-limited areas [13]. Studies have reported varying estimates of its incidence and mortality, presumably because the specific geographical regions, HEV GTs, and populations focused on in these studies were not identical. Therefore, the burden of HEV-ALF is not well understood to date. However, shedding light on the real story of HEV-ALF is the key to increasing the awareness of medical workers and promoting early diagnosis and treatment for these patients. Therefore, a systematic review and meta-analysis is necessary to comprehensively evaluate the burden of HEV-ALF in high-risk countries, namely Bangladesh, China, and India. By analyzing published eligible studies, our study aimed to summarize the currently available evidence on the burden of HEV-ALF in these three countries. The findings of this study may serve as a basis for future research on this issue.

## Methods

This systematic review and meta-analysis was registered in PROSPERO (Registration ID: CRD42022382101) and reported in accordance with the Preferred Reporting Items for Systematic Reviews and Meta-Analysis (PRISMA) [14] (Table S1).

### Data sources and searches

A comprehensive search for studies was conducted in five databases, including PubMed, the Cochrane Library, Medline, Embase, and Web of Science, from inception until 25/02/2023. The search was limited to studies published in English or Chinese. The search strategy included the keywords “HEV”, “Hepatitis E”, “liver failure”, “liver injury”, and “hepatic failure”. The detailed search strategies are provided in Table S2. In addition, a snowball tracking method was used to identify potentially relevant studies that met our inclusion criteria. All literature searches were conducted by two independent reviewers (RD and DCC). We did not contact the authors of the original studies during our study period. All eligible studies were managed in Endnote 20.0 software.

### Study selection

#### Inclusion criteria

Studies were included if they met the following criteria:

1. Studies that reported data on HEV-ALF in pregnant and non-pregnant individuals with HEV infection or HEV infection as the etiology of ALF or mortality of HEV-ALF in Bangladesh, China, and India.
2. Studies that provided explicit enough data on HEV-ALF that could be extracted for analysis.
3. Studies that stated the definition (or diagnostic criteria) of HEV infection and HEV-ALF.
4. Studies reported data on HEV-ALF in humans and focused on adults.

#### Exclusion criteria

Studies were excluded if they met the following criteria:

1. Studies were reviews, meta-analyses, case reports, randomized controlled trials, or abstracts.
2. Non-human studies.
3. Data in the studies were incomplete, insufficient, or reused.
4. Duplicate studies or full article unavailable.
5. Studies reported in neither English nor Chinese.

### Variable definitions

#### HEV infection

HEV infection was defined as the presence of anti-HEV IgM with or without the HEV-RNA confirmation [1, 15].

#### ALF and viral-related ALF

ALF was defined by the presence of jaundice, encephalopathy, and/or coagulopathy in the absence of preexisting liver disease [15]. Viral-related ALF was defined as the presence of ALF symptoms in individuals with viral infection. The common etiologies of viral-related ALF include hepatitis A virus, hepatitis B virus, hepatitis C virus, hepatitis D virus, HEV and non-A-E virus [16].

#### The proportion of HEV in the etiology of viral-related ALF

The proportion of HEV in the etiology of viral-related ALF demonstrated the proportion of HEV-ALF cases in viral-related ALF cases caused by virus infection. It was computed by dividing the number of HEV-ALF cases by the number of total viral-related ALF cases [17].

#### The prevalence of HEV-ALF in HEV-infected individuals

The prevalence of HEV-ALF in HEV-infected individuals indicated the proportion of individuals who manifested HEV-ALF among individuals with HEV infection. It was calculated as the number of HEV-ALF cases divided by the number of HEV infection cases [18].

#### The mortality of HEV-ALF

The mortality of HEV-ALF illustrated the crude death rate of participants with HEV-ALF. It was computed as the number of deceased HEV-ALF cases divided by the total HEV-ALF cases [19].

### Data extraction

A pretested data extraction form was employed in this study. Two independent reviewers (RD and DCC) extracted the necessary data from eligible studies, which were then double-checked to ensure accuracy. The extracted data included the first author’s name, year of publication, study period, study design, time of the studies, demographic characteristics of the HEV-ALF participants (age and country/region), number of HEV-ALF individuals, number of HEV-infected individuals, number of expired HEV-ALF individuals, number of viral-related ALF cases and diagnostic criteria or definition of ALF. The numbers of HEV-ALF, HEV infection, deceased HEV-ALF, and viral-related ALF cases were extracted to calculate the proportion, prevalence, and mortality. Due to the existing minor differences among the ALF definitions applied in the included studies, they were extracted to perform the subgroup analysis. Any discrepancies or disagreements between the reviewers were resolved through discussion.

### Methodological quality assessment

Methodological quality assessment of eligible studies was performed with the Joanna Briggs Institute Critical Appraisal Checklist for prevalence studies which comprised nine items in relation to risk of bias, rigor, and transparency [20] (Table S3(A)). The importance of each item was not weighted, and each item was judged with “Yes”, “No”, “Unclear”, and “Not applicable”. The number of positive items (“Yes”) a study received on a scale of nine was defined as the overall score it evaluated in the quality assessment session. Studies scored 1–3 were defined as low quality, 4–6 as moderate quality, and 7–9 as high quality. The quality evaluation was conducted by two independent reviewers (RD and DCC), and any conflicts encountered were resolved by the involvement of a third reviewer (JW).

### Statistical analysis

Pregnancy status, geographical region, and HEV GTs are crucial factors that may heavily influence the severity of HEV infection and the prognosis of HEV-ALF. Although data on HEV GTs were rarely reported in the included studies, the dominant HEV GTs varied in different countries. Therefore, the eligible studies were divided into specific groups by country and pregnancy status to estimate the burden of HEV-ALF. Statistical analysis was performed with the “metaprop” module in the R-4.0.4 statistical software package. Heterogeneity among the included studies was initially assessed with the *I*^2^ test and Cochran-Q test, in which *I*^2^ was used to indicate the percentage of variation between the included studies that was due to heterogeneity rather than sampling errors, while the presence or absence of heterogeneity was illustrated with the Cochran Q statistics. A *P* value less than 0.10 indicated the presence of heterogeneity, and the heterogeneity among studies was considered to be mild, moderate or severe if *I*^2^ was < 50%, 50–75%, and > 75%, respectively. Considering the expected heterogeneity, the estimates were calculated with DerSimonian‒Laird’s random effect models, and Freeman-Tukey double arcsine was applied before pooling data to minimize the effect of the size of study-specific estimates of rates on the overall estimate [18]. The corresponding 95% confidence interval (95% CI) was estimated utilizing the Wilson score method. Leave-one-out sensitivity analysis was conducted with the “metainf” command in the random model to identify the effect of an individual eligible study on the pooled outcome and test the reliability of the results [21]. For results only pooled data from two studies with no severe heterogeneity, we repeated the meta-analysis utilizing the fixed effect model to carry out sensitivity analysis, testing the reliability of these results [22]. In addition, subgroup analyses stratified by ALF definition were performed to determine the possible source of heterogeneity. Due to the high heterogeneity shown in most analyses, funnel plot asymmetry was not informative. Egger’s test and Begg’s test were not recommended to statistically examine the existence of publication bias in a small study sample (n < 10). Therefore, we provided the results of publication bias tests for illustration purposes only [23, 24]. Moreover, meta-regression analysis was not feasible because of the insufficient number of studies involved (n < 10) [25].

## Results

### Study selection and characteristics

**Fig. 1:**
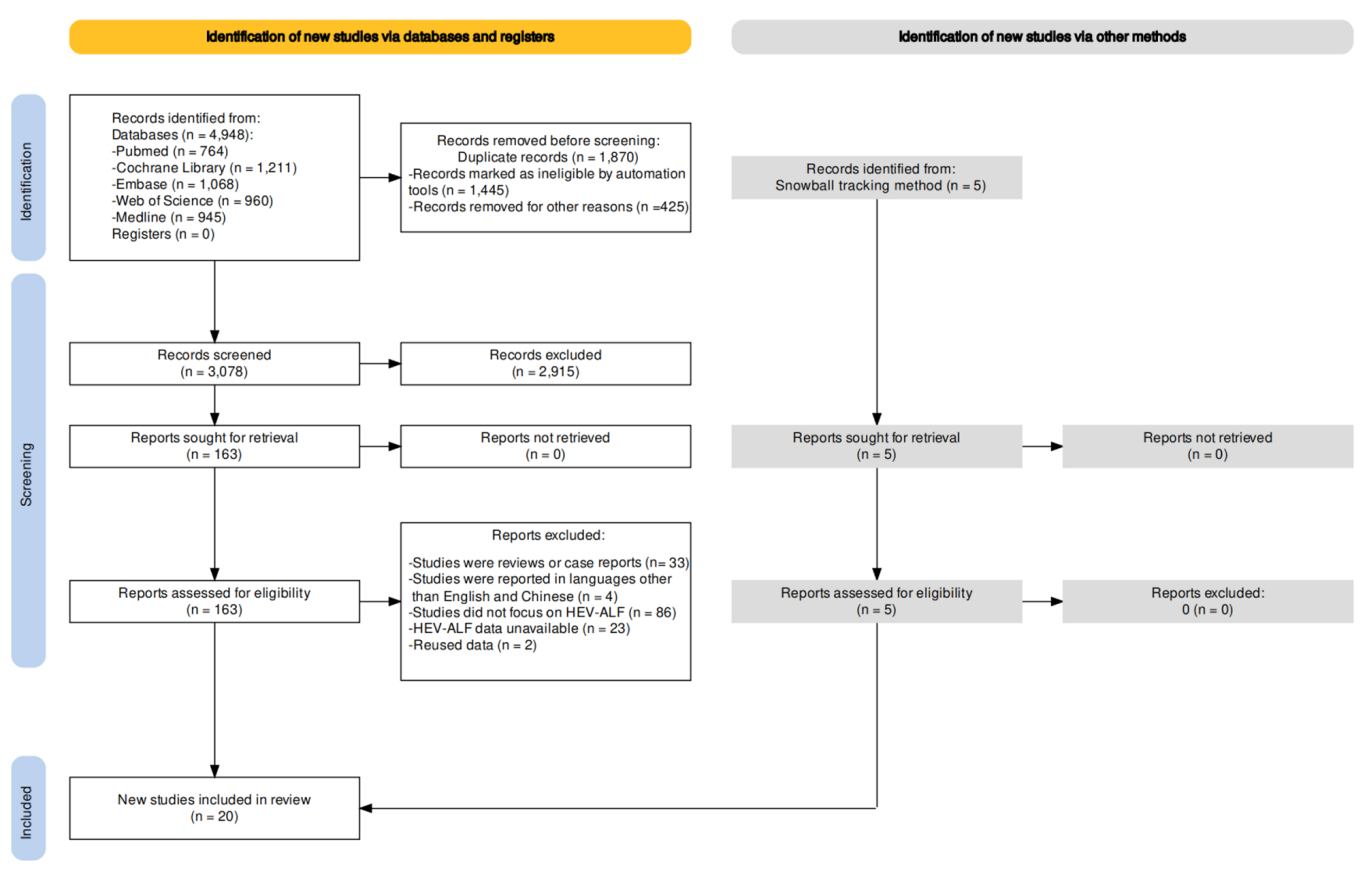
PRISMA flowchart

A comprehensive search of five databases yielded 4948 results, of which 764 were from PubMed, 1211 were from Cochrane Library, 1068 were from Embase, 960 were from Web of Science, and 945 were from Medline. Five additional candidate studies were added via the snowball tracking method. Deduplication conducted in Endnote 20.0 removed 1445 results, and 425 duplicate results were removed manually by screening their first author, titles, and abstracts. Reviewing the titles and abstracts removed 2915 candidates that did not meet our inclusion criteria. A total of 163 candidates were subjected to full-text screening for further evaluation, resulting in 20 studies meeting the inclusion criteria and being finally included in this systematic review and meta-analysis. The included studies are cited in the supplementary material. The entire selection process is depicted in Fig. 1.

The detailed characteristics of the included studies are summarized in Table 1. Of the 20 eligible studies published from 2008 to 2022, studies conducted in India accounted for the largest proportion (n = 12, 60.0%), followed by China (n = 6, 30.0%), and the other two studies were from Bangladesh. All of the included studies declared that HEV infection was diagnosed by serum anti-HEV IgM positivity and/or HEV RNA, and the definitions or diagnostic criteria for ALF are shown in Table S4. Data on HEV GTs were available in 7 eligible studies, but most of them only reported data for a limited subset of the study population. In terms of the quality assessment, 85.0% (n = 17) of the eligible studies were assessed as having moderate quality, while none of them were assessed as low quality. The detailed quality assessment information is presented in Table S3(B).

**Table 1:** Characteristic of included studies

Author, Year	Study period	Study design	Time	Center	Age	Country (Genotype)	Non-pregnant group				Pregnant group			
							A	B	C	D	A	B	C	D
Karna et al., 2020	2005-2010	cohort	P	M	15–45	India (GT1)	111	266	181	NR	102	442	139	89
You et al., 2013	2002-2011	cohort	R	S	56.1 ± 14.3	China	12	NR	50	NR	NR	NR	NR	NR
Begum et al., 2010	2006-2007	cohort	P	S	P: 23.7 ± 3.4; NP: 21.7 ± 2.9;	India (GT1)	10	31	NR	1	15	39	NR	5
Wang et al., 2019	2012-2018	nested case-control	R	S	55.15 ± 9.30	China (GT4)	35	809	NR	NR	NR	NR	NR	NR
Salam et al., 2013	2008-2011	cohort	P	S	NR	India	17	74	40	5	43	148	57	29
Asim et al., 2010	2001-2004	cohort	P	S	29.2 ± 15.7	India	21	NR	49	NR	NR	NR	NR	NR
Alam et al., 2009	2003-2008	cohort	P	M	NR	Bangladesh	33	NR	55	22	NR	NR	NR	NR
Borkakoti et al., 2013	2006-2011	cohort	P	S	P:24.6 ± 3.78; NP:26.5 ± 4.78; M:26.75 ± 15.39;	India (GT1)	58	568	170	12	105	325	160	59
Mishra et al., 2016	2013-2015	cohort	R	S	28.41 ± 10.62	India	17	62	31	8	5	NR	NR	4
Shinde et al., 2014	2008-2010	case-control	P	S	NR	India	15	44	NR	NR	24	52	NR	NR
Mu et al., 2022	2004-2020	cohort	R	M	58 (47, 64)	China	97	525	NR	33	NR	NR	NR	NR
Mahtab et al., 2009	2004-2006	cohort	R	S	27–63 (mean 39)	Bangladesh	13	NR	21	NR	NR	NR	NR	NR
Das et al.,2016	2007-2015	cohort	R	S	NR	India	34	NR	237	10	NR	NR	NR	NR
Memon et al.,2021	2019-2021	cohort	R	S	NR	India (GT1)	NR	NR	NR	NR	61	NR	100	NR
Kar et al., 2009	NR	cohort	P	S	NR	India (GT1)	11	34	50	5	29	65	50	20
Majumdar et al., 2013	2009-2012	cohort	P	S	28.38 ± 11.67	India	19	57	NR	NR	NR	NR	NR	NR
Bhatia et al., 2008	1986-2006	cohort	R	S	NR	India	197	NR	380	82	145	NR	170	74
Xiang et al., 2022	2016-2021	cohort	R	M	57.94 ± 11.35	China	200	NR	NR	42	NR	NR	NR	NR
Liu et al., 2008	2002-2007	cohort	R	M	NR	China	18	NR	48	NR	NR	NR	NR	NR
Wu et al., 2022	2018-2021	cohort	P	M	53.67 ± 12.82	China (GT3-4)	48	NR	NR	7	NR	NR	NR	NR

***Abbreviations***: P, prospective; R, retrospective; NR, not reported; GT, genotype;

***Notes***:

A: number of HEV-related acute liver failure cases;

B: number of HEV infection cases;

C: number of viral-related ALF cases;

D: number of expired HEV-ALF cases

### Meta-analysis

#### The proportion of HEV in the etiology of viral-related ALF

**Fig. 2:**
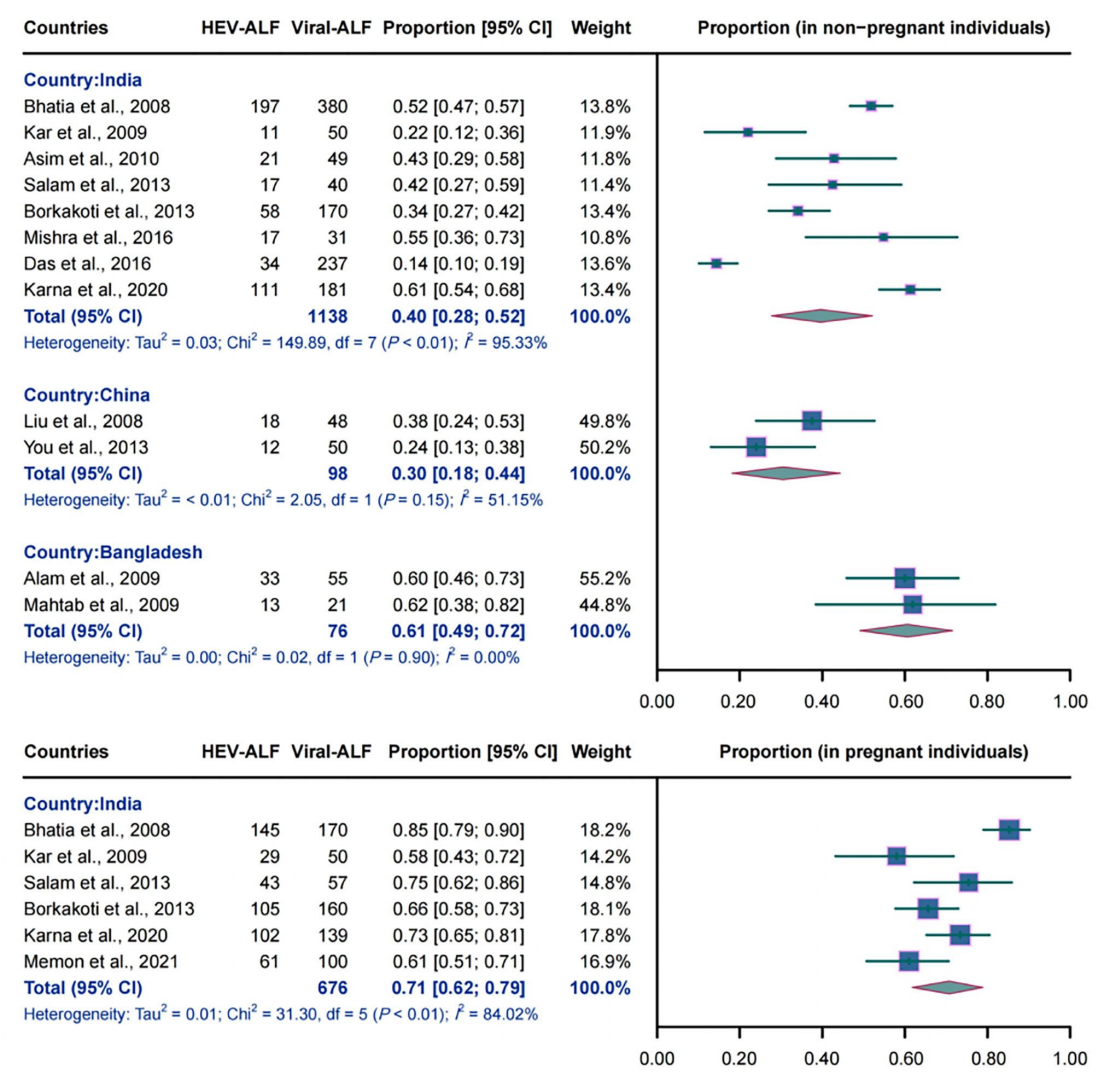
Forest plot of the pooled proportion of HEV in etiology of viral-related acute liver failure

A total of eight Indian studies (1138 non-pregnant individuals), six Indian studies (676 pregnant females), two Chinese studies (98 non-pregnant individuals), and two studies conducted in Bangladesh (76 non-pregnant participants) were pooled to estimate the proportion of HEV in etiologies of viral-related ALF. Among non-pregnant participants, the pooled proportion was estimated to be 40.0% (95% CI: 0.28–0.52, *I*^2^ = 95.33%, *P* < 0.01), 30.0% (95% CI: 0.18–0.44, *I*^2^ = 51.15%, *P* = 0.15), and 61.0% (95% CI: 0.49–0.72, *I*^2^ = 0.00%, *P* = 0.90) in India, China and Bangladesh, respectively. In pregnant Indian females, the combined proportion was 71.0% (95% CI: 0.62–0.79, *I*^2^ = 84.02%, *P* < 0.01). The detailed results are demonstrated in Fig. 2.

#### The prevalence of HEV-ALF in HEV-infected individuals

**Fig. 3:**
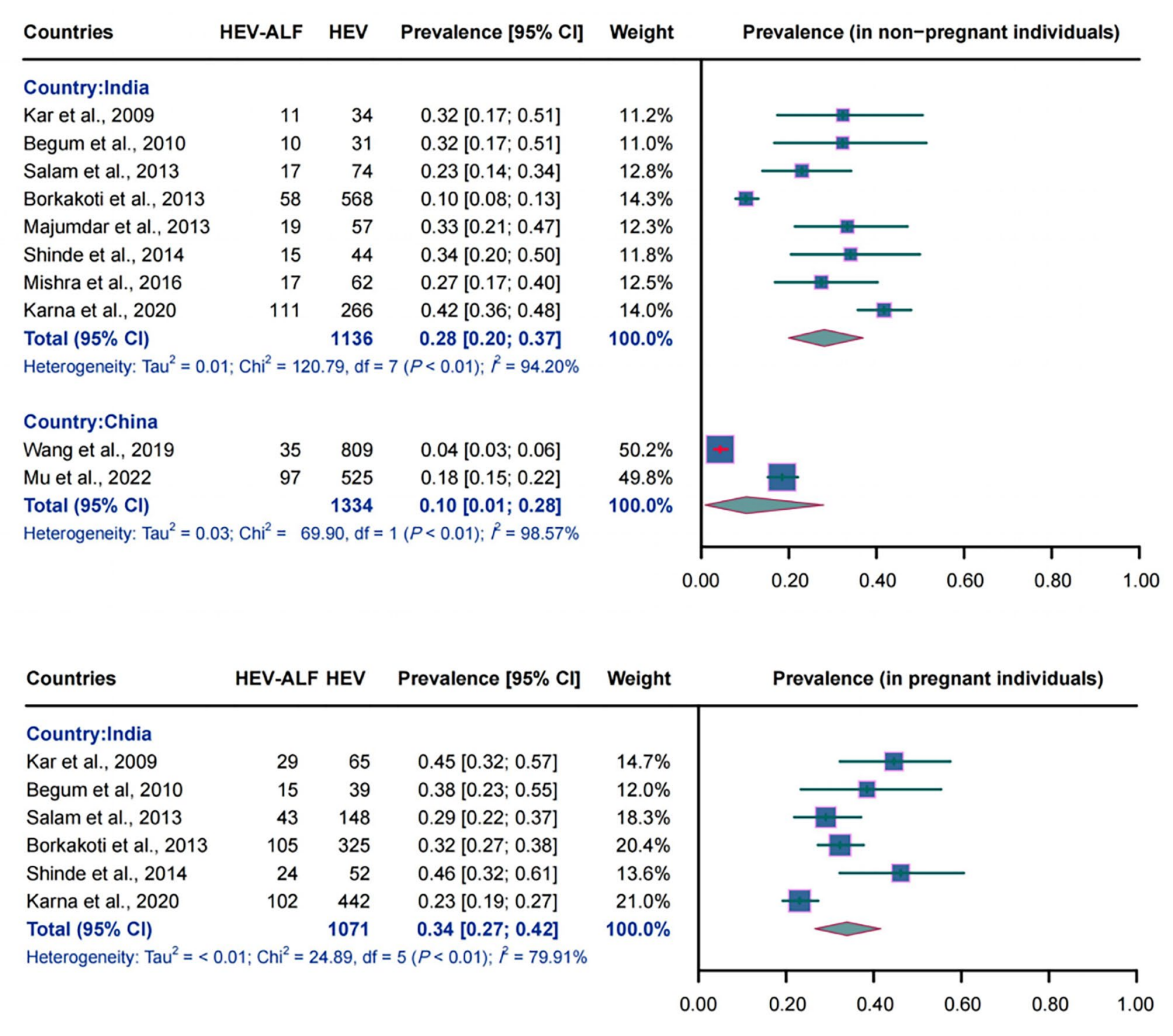
Forest plot of the combined prevalence of HEV-related acute liver failure in HEV-infected individuals

A total of eight Indian studies (1136 non-pregnant individuals), six Indian studies (1071 pregnant females), and two Chinese studies (1334 non-pregnant individuals) were pooled to estimate the prevalence of HEV-ALF among HEV-infected individuals. The combined prevalence among Indian non-pregnant HEV-infected participants was 28.0% (95% CI: 0.20–0.37, *I*^2^ = 94.20%, *P* < 0.01), while it was 34.0% (95% CI: 0.27–0.42, *I*^2^ = 79.91%, *P* < 0.01) in Indian pregnant females with HEV infection. Among Chinese non-pregnant individuals, the pooled prevalence was estimated to be 10.0% (95% CI: 0.01–0.28, *I*^2^ = 98.57%, *P* < 0.01). The detailed results are depicted in Fig. 3.

#### The mortality of HEV-ALF

**Fig. 4:**
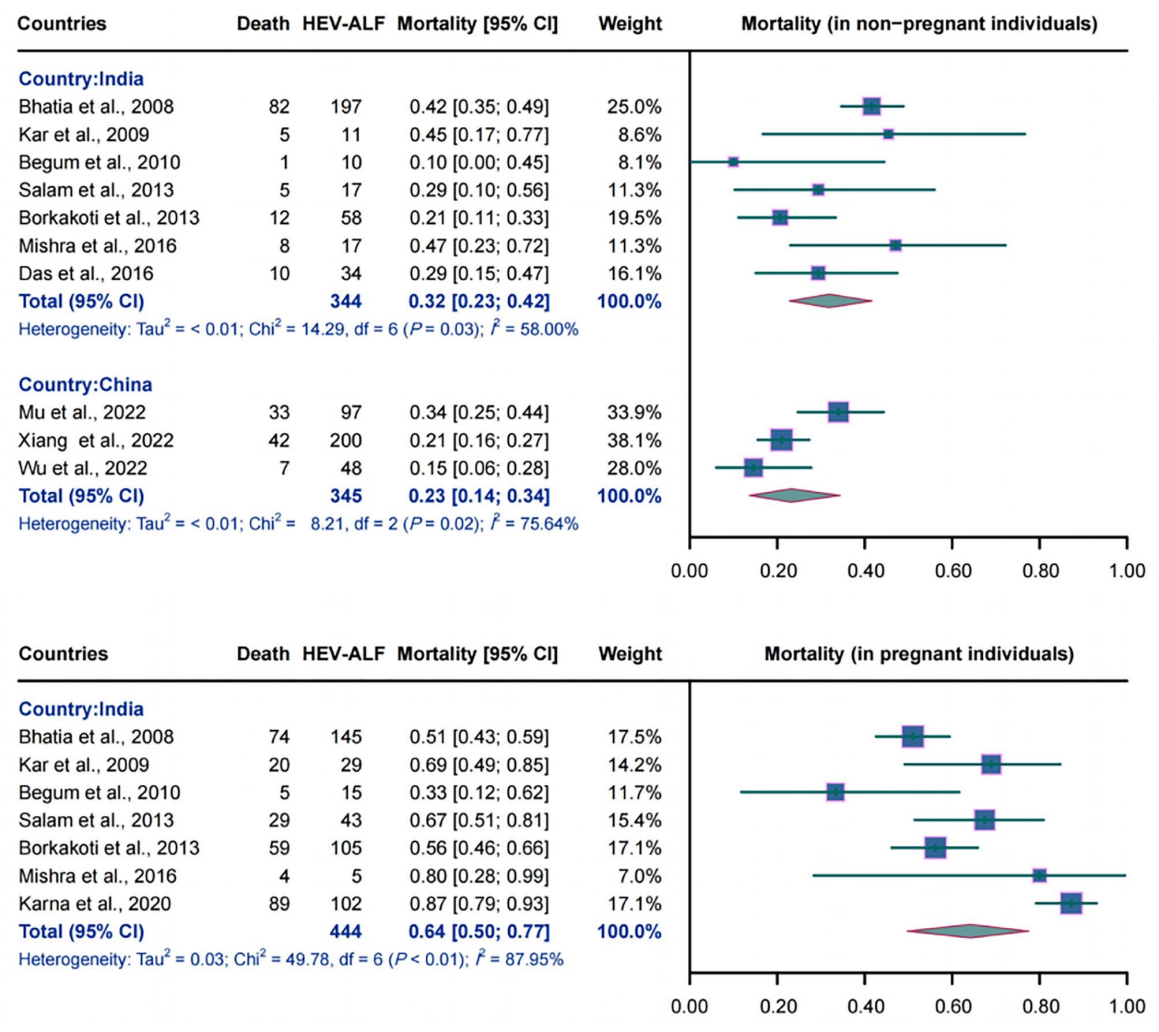
Forest plot of the pooled mortality of HEV-related acute liver failure

A total of seven Indian studies (344 non-pregnant participants), seven Indian studies (444 pregnant females), and three studies from China (345 non-pregnant individuals) were used to estimate the mortality of HEV-ALF. The overall mortality of HEV-ALF in India was 32.0% (95% CI: 0.23–0.42, *I*^2^ = 58.00%, *P* = 0.03) and 64.0% (95% CI: 0.50–0.77, *I*^2^ = 87.95%, *P* < 0.01) in the non-pregnant and pregnant participants, respectively. In China, the combined mortality of HEV-ALF among non-pregnant females was estimated to be 23.0% (95% CI: 0.14–0.34, *I*^2^ = 75.64%, *P* = 0.02). The detailed results are illustrated in Fig. 4.

### Subgroup analysis

**Fig. 5:**
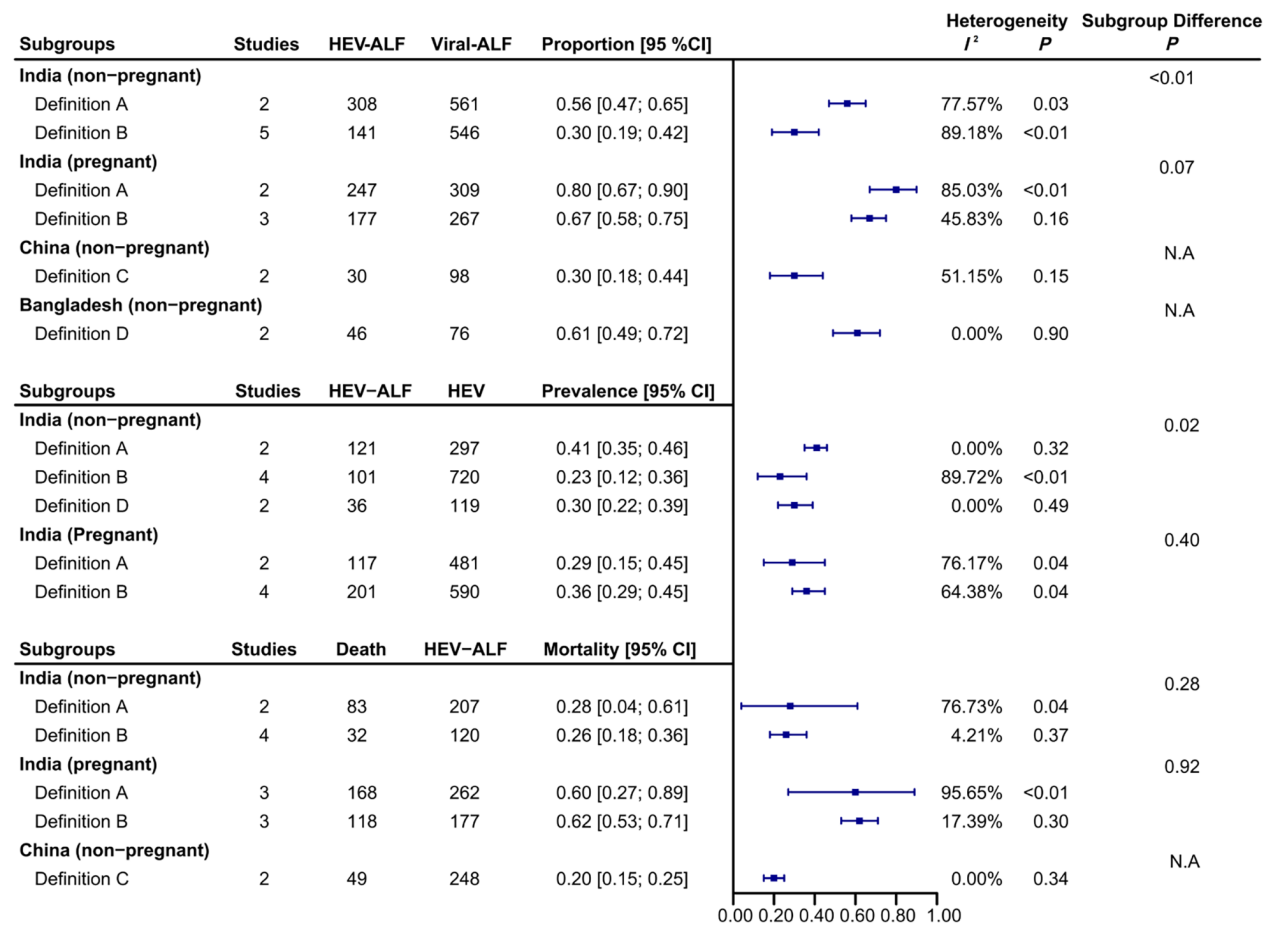
Forest plot of subgroup analysis stratified by the definition of acute liver failure

Heterogeneity was observed in most of the analyses. Therefore, the subgroup analysis stratified by the ALF definition was performed to assess the possible sources of heterogeneity. As shown in Fig. 5, the heterogeneity decreased in most of the subgroups, while some subgroups still had severe heterogeneity. All of the included studies from China and Bangladesh were in the same subgroup of ALF definition (subgroup C and subgroup D, respectively). The estimated proportion and prevalence of HEV-ALF in viral-related ALF and HEV-infected individuals showed statistical differences among ALF definition subgroups (*P*_subgroup_*<*0.05), while no statistical difference was found among pregnant females in India (*P*_subgroup_*>*0.05). However, the mortality of HEV-ALF among pregnant or non-pregnant individuals in India was similar among different subgroups of ALF definitions (*P*_subgroup_*>*0.05).

### Sensitivity analyses and publication bias tests

The leave-one-out sensitivity analysis was administered to test the reliability of the results, and the results illustrated that the pooled outcomes were not considerably changed when the studies were omitted one by one, which further indicated that the pooled results were stable (Figure S1, S2, S3). In the fixed effect model, the proportion of HEV-ALF in viral-related ALF was estimated to be 30.0% (95% CI: 0.22–0.40, *I*^2^ = 51.00%, *P* = 0.15) and 61.0% (95% CI: 0.49–0.72, *I*^2^ = 0.00%, *P* = 0.90) among non-pregnant individuals in China and Bangladesh, respectively. The combined mortality of non-pregnant participants with HEV-ALF in India was 35.0% (95% CI: 0.30–0.40, *I*^2^ = 58.00%, *P* = 0.03) (Figure S4). The results generated by a fixed effect model were generally in line with those pooled using a random effect model. Moreover, Egger’s test and Begg’s test were conducted to assess the publication bias, and the results indicated that no statistically significant publication bias was found (Table S5).

## Discussion

This systematic review and meta-analysis comprehensively estimated the burden of HEV-ALF among specific populations in three high-risk countries (Bangladesh, China, and India). We report three major findings. First, the currently available data suggested a non-negligible burden of HEV-ALF in these three countries regarding the proportion, prevalence, and mortality, especially among the specific group of pregnant females. Second, it must be noted that there were substantial variations of HEV-ALF burden in different countries and specific populations. Third, the differences in ALF definition might partly contribute to the variations in estimates of HEV-ALF burden.

Previous studies suggested that HEV is a leading cause of ALF in developing countries, especially in hyper-endemic areas, such as Bangladesh, China, and India [26, 27]. In the current study, the HEV-attributable proportion of viral-related ALF was estimated to be 40.0% in India, 30.0% in China, and 61.0% in Bangladesh among non-pregnant individuals. In addition to supporting that HEV-ALF contributes to a large share of the viral-related ALF in these countries, our study found that the HEV-attributable proportion of viral-related ALF varied across these countries. We also found the varied prevalence of HEV-ALF in HEV-infected individuals among these countries, with estimates of 28.0% in Indian non-pregnant participants and 10.0% in Chinese non-pregnant participants. The complicated characteristics of HEV epidemiology might contribute to the observed inter-country variation within these three high-burden countries. For example, the hepatic manifestations following different HEV GTs varied markedly from an entirely asymptomatic or self-limiting disease course to acute liver failure [28]. The predominant HEV GT in Bangladesh and India is HEV GT1, which is usually associated with a more severe clinical presentation, while China is prevalent with HEV GT3-4, which are considered relatively milder [28, 29]. The data on HEV GTs provided in the included studies supported this claim. Among the seven included studies that reported the data on HEV GTs, five studies from India reported HEV GT 1 infection, and two studies from China reported HEV GT 3–4 infection. The varying incidence of HEV infection in these countries also supported the observed difference [4, 5, 27]. The mortality of HEV-ALF among non-pregnant individuals was estimated to be 32.0% in India which was higher than that in China (23.0%), possibly also due to similar reasons [15, 27, 30].

The high burden and worse prognosis of HEV-ALF among Indian pregnant females are also particularly concerning. Pregnancy is a unique situation. When compared to non-pregnant individuals, pregnant females infected with HEV usually had more severe manifestations, which often present as HEV-ALF [31], especially in countries where HEV GT1-2 are endemic, causing higher mortality [26, 31, 32]. In our study, HEV was the causative agent for viral-related ALF in 71.0% of the pregnant females in India, in contrast to 40.0% of the non-pregnant individuals in the same country. The pooled prevalence of HEV-ALF was also different among non-pregnant (28.0%) and pregnant individuals (34.0%) in India. The mortality of HEV-ALF was much higher in pregnant females (64.0%) than in non-pregnant individuals (32.0%) in India. A previous meta-analysis that contained a large proportion of Indian studies also reported a similar HEV-ALF mortality of 61.2% among HEV-ALF pregnant females [33]. The existing slight difference might be due to studies from other countries where HEV GT3-4 is predominant being included in their meta-analysis. Although the detailed mechanism underlying the association between pregnancy and ALF remains unclear, current evidence shows that HEV-ALF during pregnancy is associated with hormonal changes, immunological changes, high viral loads, specific HEV GTs, and host genetic polymorphism [28, 34, 35]. Moreover, HEV infection during pregnancy was also considered to be connected with adverse fetal outcomes, including premature delivery, miscarriage, and stillbirths [29, 35]. These findings indicated that prevention of HEV infection in high-risk populations, especially pregnant females, is of great significance, and more studies are needed in the future to explore the underlying mechanisms of HEV-ALF during pregnancy.

The ALF definition was first proposed by Trey and Davidson in 1970 [36]. Over the years, several changes have been proposed, for instance, the time interval between symptoms and encephalopathy, and the geographical variation also contributed to the multiplicity of ALF definitions [1, 15, 37]. The ALF definition determines estimates of its burden and prognosis, and its variations might contribute to the observed heterogeneity [37]. Therefore, we further conducted a subgroup analysis stratified by ALF definition. The results revealed that the variations in the proportion and prevalence of HEV-ALF might be partly due to the different ALF definitions used in the included studies. Interestingly, the mortality of HEV-ALF among non-pregnant individuals or pregnant individuals in India remained similar when different ALF definitions were applied. Due to the limited number of included studies and available data, we did not perform other meaningful subgroup analyses. The age of the population and regions within one country might also contribute to the heterogeneity observed [38]. In addition, sensitivity analysis was conducted, the results of which showed that the estimates were without statistically significant changes. Moreover, no publication bias was found by Begg’s test or Egger’s test. However, due to the limited number of included studies in each specific group, the results were not informative.

Our systematic review and meta-analysis indicated that the burden of HEV-ALF in Bangladesh, China, and India is non-negligible. Necessary actions should be taken to prevent HEV infection in these high-risk countries and populations. However, the results should be treated with caution due to the limitations of our study. First, all included studies were published articles and obtained from the searched databases, the data from other resources may have an impact on the results. Second, due to the aims of our current study, the included studies were mainly focused on HEV-ALF, which might cause potential selection biases that lead to an overestimate of the HEV-ALF burden. Third, the heterogeneity ranging from moderate to severe among the included studies was not fully explained. Nevertheless, the current study represents an effort to estimate the HEV-ALF burden among specific populations in Bangladesh, China, and India based on the existing studies, and provides more information on the situation of HEV-ALF in these countries. More future large-scale studies are needed to verify these results.

## Conclusion

In conclusion, our findings suggest that the burden of HEV-ALF in Bangladesh, China, and India is non-negligible albeit with geographic and population heterogeneity. The prevention of HEV infection and early recognition of HEV-ALF are of great significance, especially in high-risk countries and populations.

### Electronic supplementary material

Below is the link to the electronic supplementary material.

Supplementary Material 1

Supplementary Material 2

Supplementary Material 3

Supplementary Material 4

Supplementary Material 5

## Data Availability

All datasets generated and/or analyzed during the current research are included in this article and its supplementary information file.

## List of Abbreviations

HEV-ALF: HEV-related acute liver failure
HEV: Hepatitis E virus
GTs: Genotypes
ALF: Acute liver failure
PRISMA: Preferred Reporting Items for Systematic Reviews and Meta-Analysis
CI: Confidence interval
