# Supplementary figures and images for: The burden of HEV-related acute liver failure in Bangladesh, China and India: a systematic review and meta-analysis
Source: BMC Public Health. 2023 Nov 29;23:2369. doi: 10.1186/s12889-023-17302-2 (PMC10688087; doi:10.1186/s12889-023-17302-2)

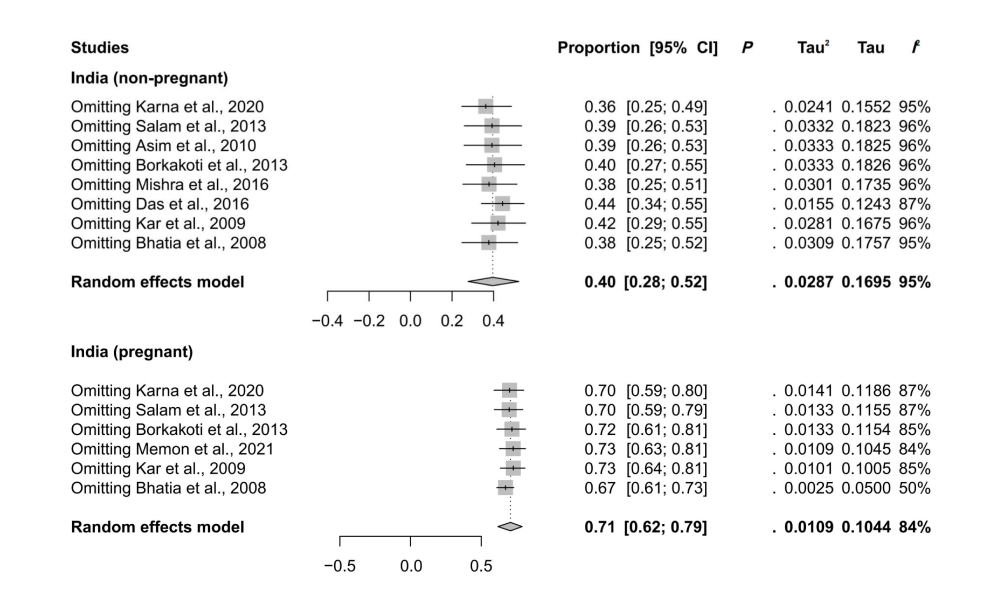

Supplement: Supplementary file 1 — Supplementary Material 1 [file 12889_2023_17302_MOESM1_ESM.png]

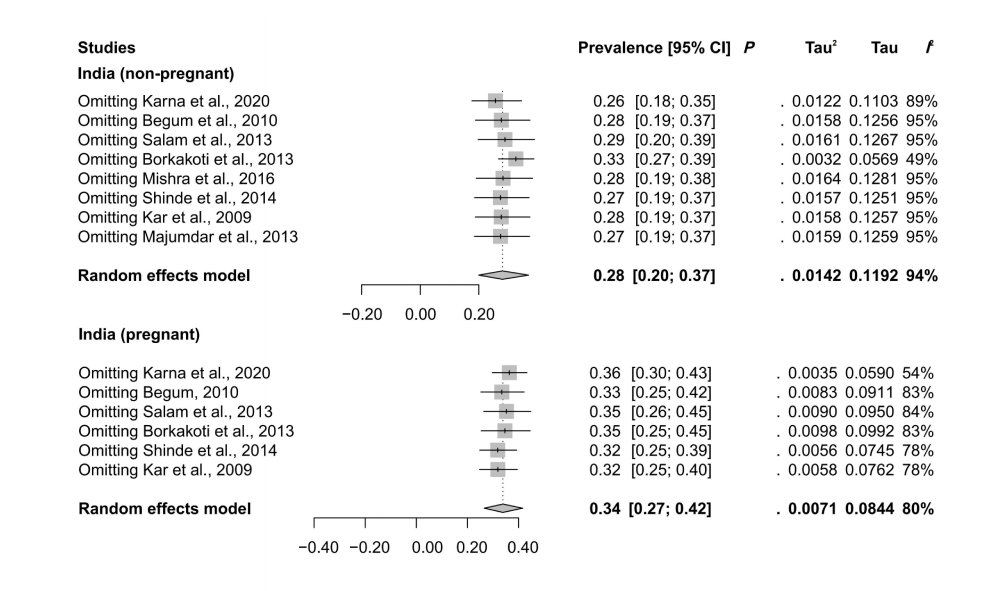

Supplement: Supplementary file 2 — Supplementary Material 2 [file 12889_2023_17302_MOESM2_ESM.png]

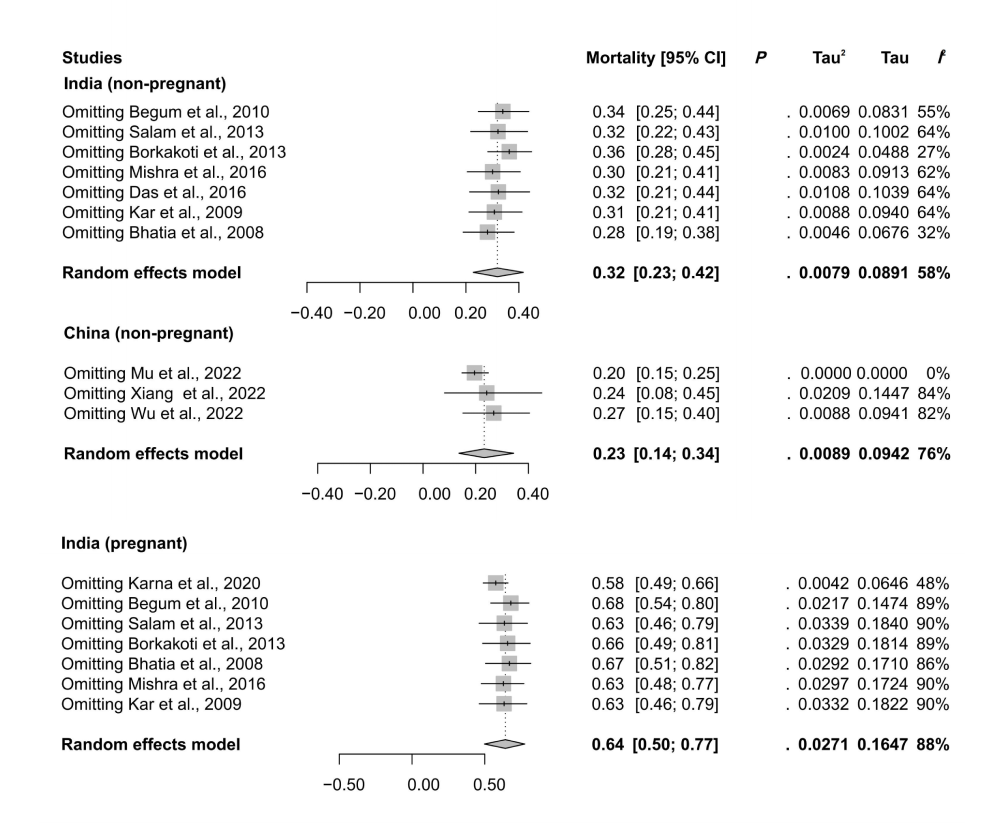

Supplement: Supplementary file 3 — Supplementary Material 3 [file 12889_2023_17302_MOESM3_ESM.png]

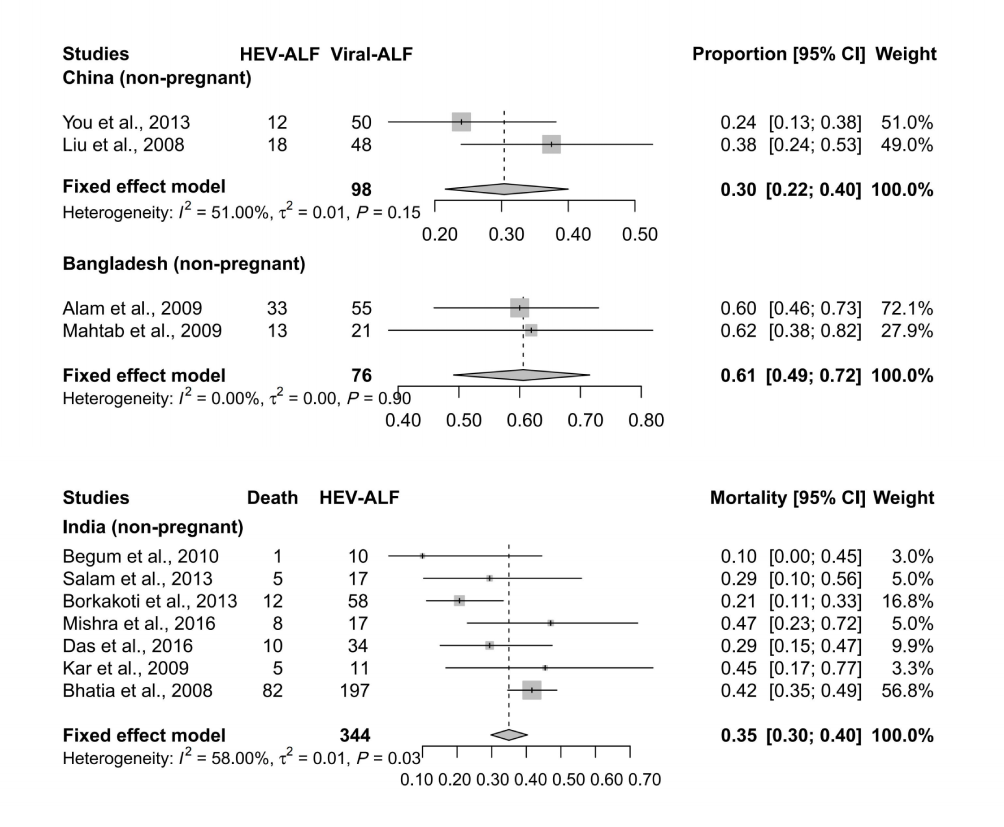

Supplement: Supplementary file 4 — Supplementary Material 4 [file 12889_2023_17302_MOESM4_ESM.png]
