# Supplementary material for: The burden of HEV-related acute liver failure in Bangladesh, China and India: a systematic review and meta-analysis
Source: BMC Public Health. 2023 Nov 29;23:2369. doi: 10.1186/s12889-023-17302-2 (PMC10688087; doi:10.1186/s12889-023-17302-2)
Supplement: Supplementary file 5 — Supplementary Material 5 [file 12889_2023_17302_MOESM5_ESM.docx]

**Supplementary Materials**

[Supplementary Table 1 PRISMA Checklist 2](#_Toc615100590)

[Supplementary Table 2 Search strategies 4](#_Toc615100590)

[Supplementary Table 3 (A) Instrument for assessment of the quality of the studies 5](#_Toc615100590)

[Supplementary Table 3 (B) The results of the quality assessment of the included studies 6](#_Toc615100590)

[Supplementary Table 4 Definitions of acute liver failure utilized in the included studies 7](#_Toc615100590)

[Supplementary Table 5 The results of publication bias tests 9](#_Toc615100590)

**[Supplementary references of the included studies](#_Toc615100590)** [1](#_Toc615100590)0

**Supplementary Table 1 PRISMA checklist**

| **Section and Topic** | **No.** | **Checklist item** | **Location where item is reported** |
| --- | --- | --- | --- |
| **TITLE** | | |  |
| Title | 1 | Identify the report as a systematic review. | Page 01 |
| **ABSTRACT** | | |  |
| Abstract | 2 | See the PRISMA 2020 for Abstracts checklist. | Page 03 |
| **INTRODUCTION** | | |  |
| Rationale | 3 | Describe the rationale for the review in the context of existing knowledge. | Page 05-06 |
| Objectives | 4 | Provide an explicit statement of the objective(s) or question(s) the review addresses. | Page 06 |
| **METHODS** | | |  |
| Eligibility criteria | 5 | Specify the inclusion and exclusion criteria for the review and how studies were grouped for the syntheses. | Page 07-08 |
| Information sources | 6 | Specify all databases, registers, websites, organisations, reference lists and other sources searched or consulted to identify studies. Specify the date when each source was last searched or consulted. | Page 07 |
| Search strategy | 7 | Present the full search strategies for all databases, registers and websites, including any filters and limits used. | Page 07 and Table S2 |
| Selection process | 8 | Specify the methods used to decide whether a study met the inclusion criteria of the review, including how many reviewers screened each record and each report retrieved, whether they worked independently, and if applicable, details of automation tools used in the process. | Page 07-08 |
| Data collection process | 9 | Specify the methods used to collect data from reports, including how many reviewers collected data from each report, whether they worked independently, any processes for obtaining or confirming data from study investigators, and if applicable, details of automation tools used in the process. | Page 09-10 |
| Data items | 10a | List and define all outcomes for which data were sought. Specify whether all results that were compatible with each outcome domain in each study were sought (e.g. for all measures, time points, analyses), and if not, the methods used to decide which results to collect. | Page 08-09 |
|  | 10b | List and define all other variables for which data were sought (e.g. participant and intervention characteristics, funding sources). Describe any assumptions made about any missing or unclear information. | Page 08-10 |
| Study risk of bias assessment | 11 | Specify the methods used to assess risk of bias in the included studies, including details of the tool(s) used, how many reviewers assessed each study and whether they worked independently, and if applicable, details of automation tools used in the process. | Page 10 |
| Effect measures | 12 | Specify for each outcome the effect measure(s) (e.g. risk ratio, mean difference) used in the synthesis or presentation of results. | Page 10-11 |
| Synthesis methods | 13a | Describe the processes used to decide which studies were eligible for each synthesis (e.g. tabulating the study intervention characteristics and comparing against the planned groups for each synthesis (item #5)). | Page 10-11 |
|  | 13b | Describe any methods required to prepare the data for presentation or synthesis, such as handling of missing summary statistics, or data conversions. | Page 10-11 |
|  | 13c | Describe any methods used to tabulate or visually display results of individual studies and syntheses. | Page 10-11 |
|  | 13d | Describe any methods used to synthesize results and provide a rationale for the choice(s). If meta-analysis was performed, describe the model(s), method(s) to identify the presence and extent of statistical heterogeneity, and software package(s) used. | Page 10-11 |
|  | 13e | Describe any methods used to explore possible causes of heterogeneity among study results (e.g. subgroup analysis, meta-regression). | Page 10-11 |
|  | 13f | Describe any sensitivity analyses conducted to assess robustness of the synthesized results. | Page 10-11 |
| Reporting bias assessment | 14 | Describe any methods used to assess risk of bias due to missing results in a synthesis (arising from reporting biases). | Page 10-11 |
| Certainty assessment | 15 | Describe any methods used to assess certainty (or confidence) in the body of evidence for an outcome. | Page 10-11 |
| **RESULTS** | | |  |
| Study selection | 16a | Describe the results of the search and selection process, from the number of records identified in the search to the number of studies included in the review, ideally using a flow diagram. | Page 11-12 |
|  | 16b | Cite studies that might appear to meet the inclusion criteria, but which were excluded, and explain why they were excluded. | Page 11-12 |
| Study characteristics | 17 | Cite each included study and present its characteristics. | Page 11-12 |
| Risk of bias in studies | 18 | Present assessments of risk of bias for each included study. | Page 11-12 |
| Results of individual studies | 19 | For all outcomes, present, for each study: (a) summary statistics for each group (where appropriate) and (b) an effect estimate and its precision (e.g. confidence/credible interval), ideally using structured tables or plots. | Page 13-14 |
| Results of syntheses | 20a | For each synthesis, briefly summarise the characteristics and risk of bias among contributing studies. | Page 13-14 |
|  | 20b | Present results of all statistical syntheses conducted. If meta-analysis was done, present for each the summary estimate and its precision (e.g. confidence/credible interval) and measures of statistical heterogeneity. If comparing groups, describe the direction of the effect. | Page 13-14 |
|  | 20c | Present results of all investigations of possible causes of heterogeneity among study results. | Page 13-14 |
|  | 20d | Present results of all sensitivity analyses conducted to assess the robustness of the synthesized results. | Page 14-15 |
| Reporting biases | 21 | Present assessments of risk of bias due to missing results (arising from reporting biases) for each synthesis assessed. | Page 14-15 |
| Certainty of evidence | 22 | Present assessments of certainty (or confidence) in the body of evidence for each outcome assessed. | Page 14-15 |
| **DISCUSSION** | | |  |
| Discussion | 23a | Provide a general interpretation of the results in the context of other evidence. | Page 16-19 |
|  | 23b | Discuss any limitations of the evidence included in the review. | Page 19-20 |
|  | 23c | Discuss any limitations of the review processes used. | Page 19-20 |
|  | 23d | Discuss implications of the results for practice, policy, and future research. | Page 19 |
| **OTHER INFORMATION** | | |  |
| Registration and protocol | 24a | Provide registration information for the review, including register name and registration number, or state that the review was not registered. | Page 06 |
|  | 24b | Indicate where the review protocol can be accessed, or state that a protocol was not prepared. | Page 06 |
|  | 24c | Describe and explain any amendments to information provided at registration or in the protocol. | Page 06 |
| Support | 25 | Describe sources of financial or non-financial support for the review, and the role of the funders or sponsors in the review. | Page 21 |
| Competing interests | 26 | Declare any competing interests of review authors. | Page 21 |
| Availability of data, code and other materials | 27 | Report which of the following are publicly available and where they can be found: template data collection forms; data extracted from included studies; data used for all analyses; analytic code; any other materials used in the review. | Page 21 |

***Reference:*** Page MJ, McKenzie JE, Bossuyt PM, et al. The PRISMA 2020 statement: An updated guideline for reporting systematic reviews. J Clin Epidemiol. 2021;134:178-189.

**Supplementary Table 2 Search Strategies**

| **Databases** | **Search Strategies** |
| --- | --- |
| Pubmed  (n=764) | (((hepatitis e[MeSH Terms]) OR (hepatitis e virus[MeSH Terms])) OR (HEV[Title/Abstract])) AND  (((((severe[Text Word]) OR (critical[Text Word])) OR (liver failure[Text Word])) OR (hepatic failure[Text Word])) OR (liver injury[Text Word]))  **Filters applied:** Humans;Chinese;English. |
| Cochrane Library  (n=1211) | #1 MeSH descriptor: [Hepatitis E] this term only  #2 hepatitis e  #3 hepatitis e virus  #4 HEV  #5 #1 OR #2 OR #3 OR #4  #6 severe  #7 critical  #8 liver failure  #9 liver injury  #10 hepatic failure  #11 hepatic injury  #12 #6 OR #7 OR #8 OR #9 OR #10 OR #11  #13 #5 AND #12  **Filter applied:** Trails; |
| Medline  (n=945) | SU=(HEV OR hepatitis e)  AND  TX=(severe OR critical OR liver injury OR liver failure OR hepatic injury OR hepatic failure)  NOT  SU=(animal)  **Filters applied:** English;Chinese; |
| Embase  (n=1068) | #1 'hepatitis e virus'/exp OR 'hepatitis e virus'  #2 hev:ti,ab  #3 #1 OR #2  #4 'liver failure'/exp OR 'liver failure'  #5 'hepatic failure'/exp OR 'hepatic failure'  #6 'liver injury'/exp OR 'liver injury'  #7 #5 OR #6  #8 #3 AND #7  **Filters applied:** Chinese;English |
| Web of Science  (n=960) | #1 (TS=(hepatitis E)) OR TS=(HEV)  #2 (((((TS=(severe)) OR TS=(critical)) OR TS=(liver failure)) OR TS=(liver injury)) OR TS=(hepatic failure)) OR TS=(hepatic injury)  #3 #1 AND #2  #4 (#3) NOT (TS=animal)  **Filters applied:** English;Chinese;Article. |

**Run date：25/02/2023**

**Supplementary Table 3 Quality assessment of included studies**

**A.Instrument for assessment of the quality of the studies**

**JBI Critical Appraisal Checklist for Studies**

**Reporting Prevalence Data**

**Reviewer:_____________ Date:____________**

**Author :_____________ Year:____________ Record Number:_______**

| **Items** | **Yes** | **No** | **Unclear** | **Not applicable** |
| --- | --- | --- | --- | --- |
| 1.Was the sample frame appropriate to address the target population? | □ | □ | □ | □ |
| 2.Were study participants sampled in an appropriate way? | □ | □ | □ | □ |
| 3.Was the sample size adequate? | □ | □ | □ | □ |
| 4.Were the study subjects and the setting described in detail? | □ | □ | □ | □ |
| 5.Was the data analysis conducted with sufficient coverage of the identified sample? | □ | □ | □ | □ |
| 6.Were valid methods used for the identification of the condition? | □ | □ | □ | □ |
| 7.Was the condition measured in a standard, reliable way for all participants? | □ | □ | □ | □ |
| 8.Was there appropriate statistical analysis? | □ | □ | □ | □ |
| 1. Was the response rate adequate?   (and if not, was the low response rate managed appropriately?) | □ | □ | □ | □ |

**Overall appraisal: Include □ Exclude□** **Seek further information□**

**Comments (Including reason for exclusion)**

**_____________________________________________________________________________________________________________________________________________________________________________________________________________________________________________________________________**

**B.The results of the quality assessment of the included studies**

| **Author**  **Year** | **Individual Items** | | | | | | | | | **Overall**  **score** |
| --- | --- | --- | --- | --- | --- | --- | --- | --- | --- | --- |
|  | **Item 1** | **Item 2** | **Item 3** | **Item 4** | **Item 5** | **Item 6** | **Item 7** | **Item 8** | **Item 9** |  |
| Karna, 2019 |  | ★ | ★ | ★ |  |  | ★ |  |  | 4(moderate quality) |
| You, 2013 | ★ |  | ★ | ★ |  | ★ |  |  | ★ | 5(moderate quality) |
| Begum, 2010 | ★ |  | ★ |  |  | ★ | ★ | ★ |  | 5(moderate quality) |
| Wang, 2019 | ★ |  | ★ | ★ |  | ★ |  | ★ | ★ | 6(moderate quality) |
| Salam, 2013 | ★ |  | ★ | ★ |  | ★ | ★ | ★ |  | 6(moderate quality) |
| Asim, 2008 | ★ |  | ★ |  | ★ | ★ |  | ★ | ★ | 6(moderate quality) |
| Alam, 2008 | ★ |  | ★ | ★ | ★ | ★ |  | ★ |  | 6(moderate quality) |
| Borkakoti, 2013 | ★ |  | ★ |  | ★ |  | ★ | ★ |  | 5(moderate quality) |
| Mishra, 2016 |  |  | ★ |  |  | ★ | ★ | ★ |  | 4(moderate quality) |
| Shinde, 2014 | ★ |  | ★ | ★ |  |  | ★ | ★ | ★ | 6(moderate quality) |
| Mu, 2020 | ★ |  | ★ |  | ★ | ★ | ★ | ★ |  | 6(moderate quality) |
| Mahtab,2009 | ★ |  | ★ |  | ★ |  | ★ |  | ★ | 5(moderate quality) |
| Das, 2016 | ★ | ★ | ★ |  |  |  | ★ |  | ★ | 5(moderate quality) |
| Memon, 2021 | ★ |  | ★ | ★ |  | ★ | ★ | ★ | ★ | 7(high quality) |
| Kar, 2009 | ★ |  | ★ | ★ |  | ★ | ★ | ★ | ★ | 7(high quality) |
| Bhatia,2013 | ★ |  | ★ | ★ |  |  | ★ | ★ | ★ | 6(moderate quality) |
| Majumdar,2013 |  |  | ★ | ★ |  |  |  | ★ | ★ | 4(moderate quality) |
| Xiang, 2022 | ★ |  | ★ | ★ | ★ | ★ |  | ★ | ★ | 7(high quality) |
| Liu, 2008 |  |  | ★ | ★ | ★ |  |  | ★ |  | 4(moderate quality) |
| Wu, 2022 | ★ |  | ★ | ★ |  |  | ★ | ★ |  | 5(moderate quality) |

**Supplementary Table.4 Definitions of acute liver failure utilized in the included studies**

| **Studies** | **Definitions or diagnostic criteria of acute liver failure (ALF)** | **Subgroup** |
| --- | --- | --- |
| Karna et al, 2020 | ALF was defined as the patients, with no known pre-existing liver disease, had hepatic encephalopathy within 4 weeks of the onset of disease. | A |
| You et al, 2013 | ALF is defined as the presence of coagulopathy (prothrombin activity [PTA] ≤40%) and hepatic encephalopathy (at least grade2) within 2 weeks of the first symptoms without previous underlying liver disease. | C |
| Begum et al, 2010 | ALF was defined as the patients, with no known pre-existing liver disease, had hepatic encephalopathy within 4 weeks of the onset of disease. | A |
| Wang et al, 2019 | Diagnosis of ALF is based on the presence of stage 2 or 3 encephalopathy complicating end-stage disease manifestations, including profound coagulopathy (PTA ≤40% or international normalized ratio [INR] ≥1.5), jaundice and hepatic atrophy in two weeks in patients with no chronic liver disease (CLD)； | C |
| Salam et al, 2013 | ALF was diagnosed when after a typical acute onset, the patient became deeply jaundiced and went into hepatic encephalopathy within 8 weeks of onset of the disease without any past history of CLD. | B |
| Asim et al, 2010 | ALF was considered when after a typically acute onset, the patient become deeply jaundiced and went into hepatic encephalopathy within 8 weeks of onset of the disease, with no past history of CLD. | B |
| Alam et al, 2009 | The diagnosis of FHF was based on the occurrence of hepatic encephalopathy within 8 weeks of onset of jaundice in patients with no previous liver disease and the presence of coagulopathy as proved by a prothrombin time (PT) > 15 s or INR > 1.5. | D |
| Borkakoti et al, 2013 | ALF was considered when after a typically acute onset, the patient become deeply jaundiced and went into hepatic encephalopathy within 8 weeks of onset of the disease, with no past history of CLD. | B |
| Mishra et al, 2016 | Suspected cases of ALF were defined as: any evidence of coagulation abnormality, generally an INR >1.5 and any degree of mental alteration (encephalopathy) without pre-existing cirrhosis and with an illness of < 4 weeks’ duration. | D |
| Shinde et al, 2014 | ALF was considered when after a typically acute onset, the patient become deeply jaundiced and went into hepatic encephalopathy within 8 weeks of onset of the disease, with no past history of CLD. | B |
| Mu et al, 2022 | ALF was defined as patients who were hospitalized with PT values of 40% or less of the standardized value, or INR of 1.5 or more due to severe liver damage within 8 weeks of the first symptoms, and mentation in the absence of CLD. | D |
| Mahtab et al, 2009 | Patients were diagnosed based on the history from attendants, clinical features, physical examinations, laboratory investigations, and imaging. Only those patients who presented with severe impairment of hepato-cellular function, i.e. encephalopathy, coagulopathy, and jaundice, within six months of onset of symptoms were included in the study. They were all previously healthy. | D |
| Das et al, 2016 | ALF was considered when after a typically acute onset, the patient become deeply jaundiced and went into hepatic encephalopathy within 8 weeks of onset of the disease, with no past history of CLD. | B |
| Memon et al, 2021 | Patients with no known CLD presented as hepatic encephalopathy, jaundice and coagulopathy were labeled as ALF. | D |
| Kar et al, 2009 | ALF was diagnosed when after a typical acute onset, patient become deeply jaundiced and went into hepatic encephalopathy within 8 week of onset of disease, without any past history of CLD. | B |
| Majumdar et al, 2013 | ALF was considered when the patient develop evidence of coagulation abnormality with an INR ≥ 1.5 and develop encephalopathy with an illness of <26 weeks duration, without any history of pre-existing liver disease. | D |
| Bhatia et al, 2008 | ALF was defined by the occurrence of encephalopathy within 4 weeks of onset of symptoms in the absence of pre-existing liver disease. | A |
| Xiang et al, 2022 | ALF was diagnosed as followed: abnormal liver synthetic function, including PTA of less than 40% combined with hepatic atrophy and jaundice in 2 weeks; encephalopathy in stage 2/3 complicated with manifestations of end‐stage disease; no CLD. | C |
| Liu et al, 2008 | ALF was defined based on the presence of stage 2 or 3 encephalopathy complicating end-stage disease manifestations, including profound coagulopathy (PTA ≤40% or INR ≥1.5), jaundice and hepatic atrophy in two weeks in patients with no CLD； | C |
| Wu et al, 2022 | ALF was defined as showing evidence of abnormal liver synthetic function (PTA ≤40% or INR ≥1.5), jaundice, and hepatic atrophy over a 2-week period; the presence of stage 2 or 3 encephalopathy complicating end-stage disease manifestations with no CLD. | C |

***Notes:*** Studies in subgroup A directly applied or used the same ALF definition proposed by International Association for the Study of the Liver Subcommittee^[1]^, including Karna et al., 2020, Begum et al., 2010 and Bhatia et al., 2008; Studies in subgroup B utilized the ALF definition proposed by Trey and Davidson^[2]^, including Salam et al., 2013, Asim et al., 2010, Borkakoti et al., 2013, Shinde et al., 2014, Das et al., 2016 and Kar et al., 2009; Studies in subgroup C defined ALF based on the Chinese diagnostic and treatment guidelines for liver failure^[3]^, or used the same ALF definition, including You et al., 2013, Wang et al., 2019, Xiang et al., 2022; Wu et al., 2022 and Liu et al., 2008; Studies in subgroup D utilized ALF definitions other than subgroup A-C, but they could not be further classified into subgroups that contained ≥ 2 studies, including Alam et al, 2009, Mishra et al, 2016, Mu et al, 2022, Mahtab et al, 2009; Memon et al, 2021 and Majumdar et al, 2013.

***References:***

1. Tandon BN, Bernauau J, O'Grady J, et al. Recommendations of the International Association for the Study of the Liver Subcommittee on nomenclature of acute and subacute liver failure. J Gastroenterol Hepatol. 1999;14(5):403-404.

[2]Trey C, Davidson CS. The management of fulminant hepatic failure. Prog Liver Dis. 1970;3:282-298.

[3]Liver Failure and Artificial Liver Group, Chinese Society of Infectious Diseases, Chinese Medical Association; Severe Liver Diseases and Artificial Liver Group, Chinese Society of Hepatology, Chinese Medical Association. Diagnostic and treatment guidelines for liver failure (2012 version). Zhonghua Gan Zang Bing Za Zhi. 2013;21(3):177–183.

**Supplementary Table.5 The results of publication bias tests**

| **Results** | **Groups** | ***P* value**  **(Begg’s test)** | ***P* value**  **(Egger’s test)** |
| --- | --- | --- | --- |
| Proportion of HEV in the etiology of viral-related ALF | India (non-pregnant participants) | 0.80 | 0.18 |
|  | India (pregnant participants) | 0.35 | 0.38 |
|  | China (non-pregnant participants) | N.A | N.A |
|  | Bangladesh (non-pregnant participants) | N.A | N.A |
| Prevalence of HEV-ALF in HEV infected individuals | India (non-pregnant participants) | 0.62 | 0.17 |
|  | India (pregnant participants) | 0.35 | 0.05 |
|  | China (non-pregnant participants) | N.A | N.A |
| Mortality of HEV-ALF | India (non-pregnant participants) | 0.76 | 0.38 |
|  | India (pregnant participants) | 0.29 | 0.99 |
|  | China (non-pregnant participants) | N.A | N.A |

***Notes:*** Technically, the minimum number of studies is three to conduct Begg’s test and Egger’s test using “metabias” command in R package. When the sample size is less than three studies, it is not possible to do Begg’s test and Egger’s test, the results were labeled as “N.A”. However, these tests should only be conducted if the number of studies is ten or larger following recommendations^[1]^.

**References:**

[1]Sterne JA, Sutton AJ, Ioannidis JP, et al. Recommendations for examining and interpreting funnel plot asymmetry in meta-analyses of randomised controlled trials. BMJ. 2011;343:d4002.

**Supplementary references of included studies**

The following studies were included in the systematic review and meta-analysis

[1] Karna R, Hazam RK, Borkakoti J, Kumar A, Kar P. A 5-year Single-Center Experience of Hepatitis E Virus Infection During Pregnancy. J Clin Exp Hepatol. 2020;10(2):135-138. doi:10.1016/j.jceh.2019.09.003

[2] You S, Rong Y, Zhu B, et al. Changing etiology of liver failure in 3,916 patients from northern China: a 10-year survey. Hepatol Int. 2013;7(2):714-720. doi:10.1007/s12072-013-9424-5

[3] Begum N, Polipalli SK, Husain SA, Kumar A, Kar P. Duration of hepatitis E viremia in pregnancy. Int J Gynaecol Obstet. 2010;108(3):207-210. doi:10.1016/j.ijgo.2009.09.023

[4] Wang Y, Liu H, Liu S, et al. Incidence, predictors and prognosis of genotype 4 hepatitis E related liver failure: A tertiary nested case-control study. Liver Int. 2019;39(12):2291-2300. doi:10.1111/liv.14221

[5] Salam GD, Kumar A, Kar P, Aggarwal S, Husain A, Sharma S. Serum tumor necrosis factor-alpha level in hepatitis E virus-related acute viral hepatitis and fulminant hepatic failure in pregnant women. Hepatol Res. 2013;43(8):826-835. doi:10.1111/hepr.12028

[6] Asim M, Singla R, Gupta RK, Kar P. Clinical & molecular characterization of human TT virus in different liver diseases. Indian J Med Res. 2010;131:545-554.

[7] Alam S, Azam G, Mustafa G, et al. Natural course of fulminant hepatic failure: the scenario in Bangladesh and the differences from the west. Saudi J Gastroenterol. 2009;15(4):229-233. doi:10.4103/1319-3767.56094

[8] Borkakoti J, Hazam RK, Mohammad A, Kumar A, Kar P. Does high viral load of hepatitis E virus influence the severity and prognosis of acute liver failure during pregnancy?. J Med Virol. 2013;85(4):620-626. doi:10.1002/jmv.23508

[9] Mishra S, Borkakoti J, Kumar S, Kar P. Role of HEV antigen detection in HEV-related acute viral hepatitis and acute liver failure. J Med Virol. 2016;88(12):2179-2185. doi:10.1002/jmv.24567

[10] Shinde N, Patil T, Deshpande A, Gulhane R, Patil M, Bansod Y. Clinical profile, maternal and fetal outcomes of acute hepatitis e in pregnancy. Ann Med Health Sci Res. 2014;4(Suppl 2):S133-S139. doi:10.4103/2141-9248.138033

[11] Mu X, Zou J, Chen J, et al. Low platelets: a new and simple prognostic marker for patients with hepatitis E virus-related acute liver failure. Hepatol Int. 2022;16(5):1116-1126. doi:10.1007/s12072-022-10302-1

[12] Mamun-Al-Mahtab, Rahman S, Khan M, Karim F. HEV infection as an aetiologic factor for acute hepatitis: experience from a tertiary hospital in Bangladesh. J Health Popul Nutr. 2009;27(1):14-19. doi:10.3329/jhpn.v27i1.3314

[13] Das AK, Begum T, Kar P, Dutta A. Profile of Acute Liver Failure from North-east India and Its Differences from other Parts of the Country. Euroasian J Hepatogastroenterol. 2016;6(2):111-115. doi:10.5005/jp-journals-10018-1181

[14] Memon FP, Memon SA, Almas. et al. Fulminant Hepatic Failure in Pregnancy and its Association with Viral Hepatitis E. Medical Forum Monthly. 2021;32(11):133-136

[15] Kar P, Jilani N, Husain SA, et al. Does hepatitis E viral load and genotypes influence the final outcome of acute liver failure during pregnancy?. Am J Gastroenterol. 2008;103(10):2495-2501. doi:10.1111/j.1572-0241.2008.02032.x

[16] Majumdar M, Ratho R, Chawla Y, Singh MP. High levels of circulating HMGB1 as a biomarker of acute liver failure in patients with viral hepatitis E. Liver Int. 2013;33(9):1341-1348. doi:10.1111/liv.12197

[17] Bhatia V, Singhal A, Panda SK, Acharya SK. A 20-year single-center experience with acute liver failure during pregnancy: is the prognosis really worse?. Hepatology. 2008;48(5):1577-1585. doi:10.1002/hep.22493

[18] Xiang Z, Jiang B, Li W, et al. The diagnostic and prognostic value of serum exosome-derived carbamoyl phosphate synthase 1 in HEV-related acute liver failure patients. J Med Virol. 2022;94(10):5015-5025. doi:10.1002/jmv.27961

[19] Liu XY, Hu JH, Wang HF, Chen JM. Etiological analysis of 1977 patients with acute liver failure, subacute liver failure and acute-on-chronic liver failure. Zhonghua Gan Zang Bing Za Zhi. 2008;16(10):772-775.

[20] Wu J, Xu Y, Cui Y, et al. Dynamic changes of serum metabolites associated with infection and severity of patients with acute hepatitis E infection. J Med Virol. 2022;94(6):2714-2726. doi:10.1002/jmv.27669
